# Prevalence and characteristics of patients with incidental cardiac uptake on bone scintigraphy

**DOI:** 10.1186/s44348-024-00030-5

**Published:** 2024-08-03

**Authors:** Jihee Son, Yeon-Hee Han, Sun Hwa Lee

**Affiliations:** 1 Division of Cardiology, Department of Internal Medicine, Jeonbuk National University Medical School, Jeonju, Republic of Korea; 2 Research Institute of Clinical Medicine of Jeonbuk National University, Biomedical Research Institute of Jeonbuk National University Hospital, Jeonju, Republic of Korea; 3 Department of Nuclear Medicine, Cyclotron Research Center, Molecular Imaging and Therapeutic Medicine Research Center, Jeonbuk National University HospitalJeonbuk National University Medical School, Jeonju, Republic of Korea

**Keywords:** Amyloidosis, Cardiac, Radionuclide imaging, Prevalence

## Abstract

**Background:**

Bone scintigraphy is emerging as a confirmatory diagnostic tool for transthyretin cardiac amyloidosis (ATTR-CA). This study aimed to investigate the frequency and clinical characteristics of patients with incidental cardiac uptake and incidental ATTR-CA on bone scintigraphy.

**Methods:**

All bone scintigraphic studies performed at a tertiary teaching hospital between 2011 and 2022 were reviewed retrospectively. Patients who underwent bone scintigraphy to confirm ATTR-CA were excluded. Patients with cardiac uptake of grade 2 or 3 were included and divided into two groups: possible ATTR-CA group and noncardiac amyloidosis (non-CA) group.

**Results:**

Of the 61,432 bone scintigraphic studies performed on 32,245 patients, 23 (0.07%) had grade 2 or 3 cardiac uptake. Nine of 23 patients (39.1%) were assigned to the non-CA group because they showed cardiac uptake from definite other causes or focal uptake that did not match CA. The remaining 14 patients (60.9%) were classified as the possible ATTR-CA group, and five patients were referred to cardiologists and finally diagnosed with ATTR-CA. Two patients were treated with tafamidis. Patients in the ATTR-CA group were significantly older and had a less frequent history of end-stage renal disease than those in the non-CA group. Other characteristics were comparable in both groups.

**Conclusions:**

Although incidental ATTR-CA in patients undergoing bone scintigraphy for noncardiac reasons is uncommon, if cardiac uptake is observed in elderly patients without metastatic calcification associated with end-stage renal disease, further diagnostic work-up for ATTR-CA as a cause of undiagnosed heart failure should be considered.

## Background

Transthyretin cardiac amyloidosis (ATTR-CA) is an infiltrative and restrictive cardiomyopathy caused by the extracellular deposition of insoluble transthyretin amyloid fibers in the myocardium [1]. Wild-type ATTR-CA (wtATTR-CA) is a senile disease that predominantly affects men, primarily involving the heart, and may be asymptomatic in the early stages, but as it progresses, heart failure (HF), arrhythmias, and conduction abnormalities develop and worsen, finally leading to death [1, 2].

The clinical manifestations of ATTR-CA are nonspecific HF symptoms such as dyspnea or edema, which can take years or longer from the first HF symptoms to diagnosis [3], and orthopedic symptoms such as carpal tunnel syndrome or neuropathy, which are caused by transthyretin amyloid deposition in soft tissue and can precede HF symptoms by up to 10 years [4, 5], contributing to the delay in diagnosis of ATTR-CA.

With the advent of a new era of disease-modifying therapies for ATTR-CA, it is widely recognized that early detection of the disease and initiation of treatment before the onset of cardiac dysfunction is associated with a better prognosis for ATTR-CA [6]. There is also growing interest in nonbiopsy ATTR-CA diagnosis, as bone scintigraphy, which has been used clinically primarily for orthopedic or oncologic purposes, can detect ATTR-CA in the asymptomatic stage before changes in the electrocardiogram (ECG) or echocardiography become apparent [7, 8]. This study aimed to investigate the prevalence of incidentally diagnosed ATTR-CA in patients by bone scintigraphy undergoing orthopedic or oncologic purposes without symptoms of HF and the clinical characteristics of those patients.

## Methods

### Subjects

We retrospectively reviewed all consecutive bone scintigraphic images obtained at Jeonbuk National University Hospital (Jeonju, Republic of Korea) between 2011 and 2022. A total of 61,432 bone scintigraphic images from 32,245 patients were analyzed. Bone scintigraphic images included bone scans and single-photon emission computed tomography (SPECT) studies acquired utilizing technetium-99m–labeled 3,3-diphosphono-1,2-propanodicarboxylic acid (^99m^Tc-DPD) or technetium-99m–labeled hydroxymethylene diphosphonate (^99m^Tc-HMDP).

**Fig. 1:**
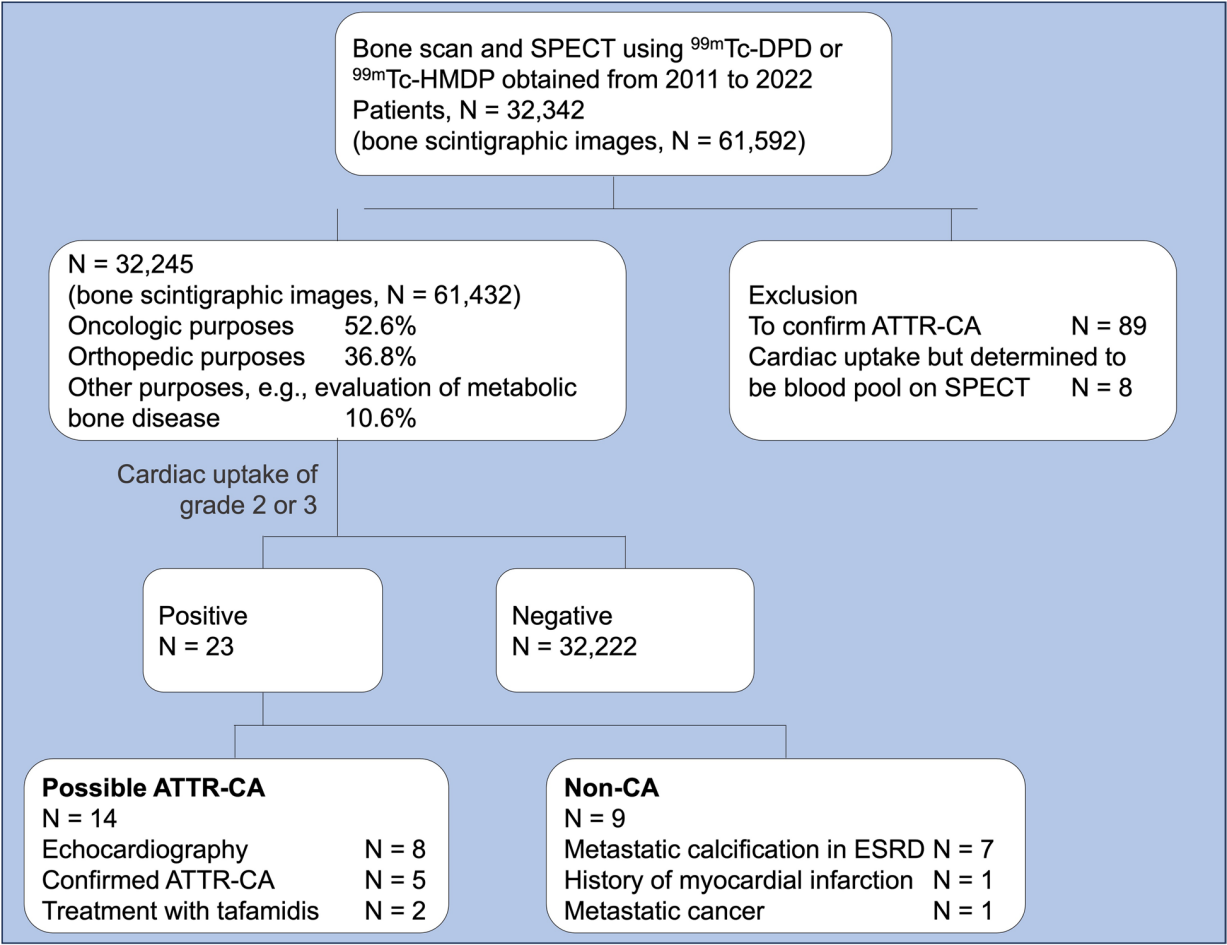
Flowchart showing the number of patients excluded, the reasons for exclusion, and the two groups included in this study. SPECT, single-photon emission computed tomography; ^99m^Tc-DPD, technetium-99m–labeled 3,3-diphosphono-1,2-propanodicarboxylic acid; ^99m^Tc-HMDP, technetium-99m–labeled hydroxymethylene diphosphonate; ATTR-CA, transthyretin cardiac amyloidosis; ESRD, end-stage renal disease; CA, cardiac amyloidosis

Nuclear imaging specialists originally interpreted the bone scintigraphic images, and in cases with cardiac uptake, two cardiac imaging specialists retrospectively verified the interpretation. Diffuse cardiac uptake of Perugini grade 2 or 3 was considered as a possible ATTR-CA [9]. Patients who underwent bone scintigraphy to confirm ATTR-CA were excluded. Patients initially classified as having cardiac uptake were excluded if a comparison with corresponding SPECT images revealed that the uptake was in the blood pool rather than the myocardium (Fig. 1).

### Clinical and laboratory data

The baseline clinical characteristics including age, sex, cardiovascular risk factors such as diabetes mellitus and hypertension, history of end-stage renal disease (ESRD), HF, and cancer, and ECG and laboratory findings of patients with unexpected cardiac uptake of grade 2 or greater were collected by the review of medical records. In patients who underwent echocardiography, the parameters of left ventricular ejection fraction (LVEF), global longitudinal strain (GLS), left ventricular (LV) wall thickness, size of the left atrium, and diastolic function were measured.

### Bone scintigraphic assessment

Bone scan or SPECT was obtained according to the standard protocol. Whole-body imaging and localized imaging in the anterior and posterior views were acquired 3 h after intravenous injection of 30 mCi of bone tracer, ^99m^Tc-DPD or ^99m^Tc-HMDP. The type of tracers used was determined by the laboratory’s policy at the time the scintigraphy was performed.

**Fig. 2:**
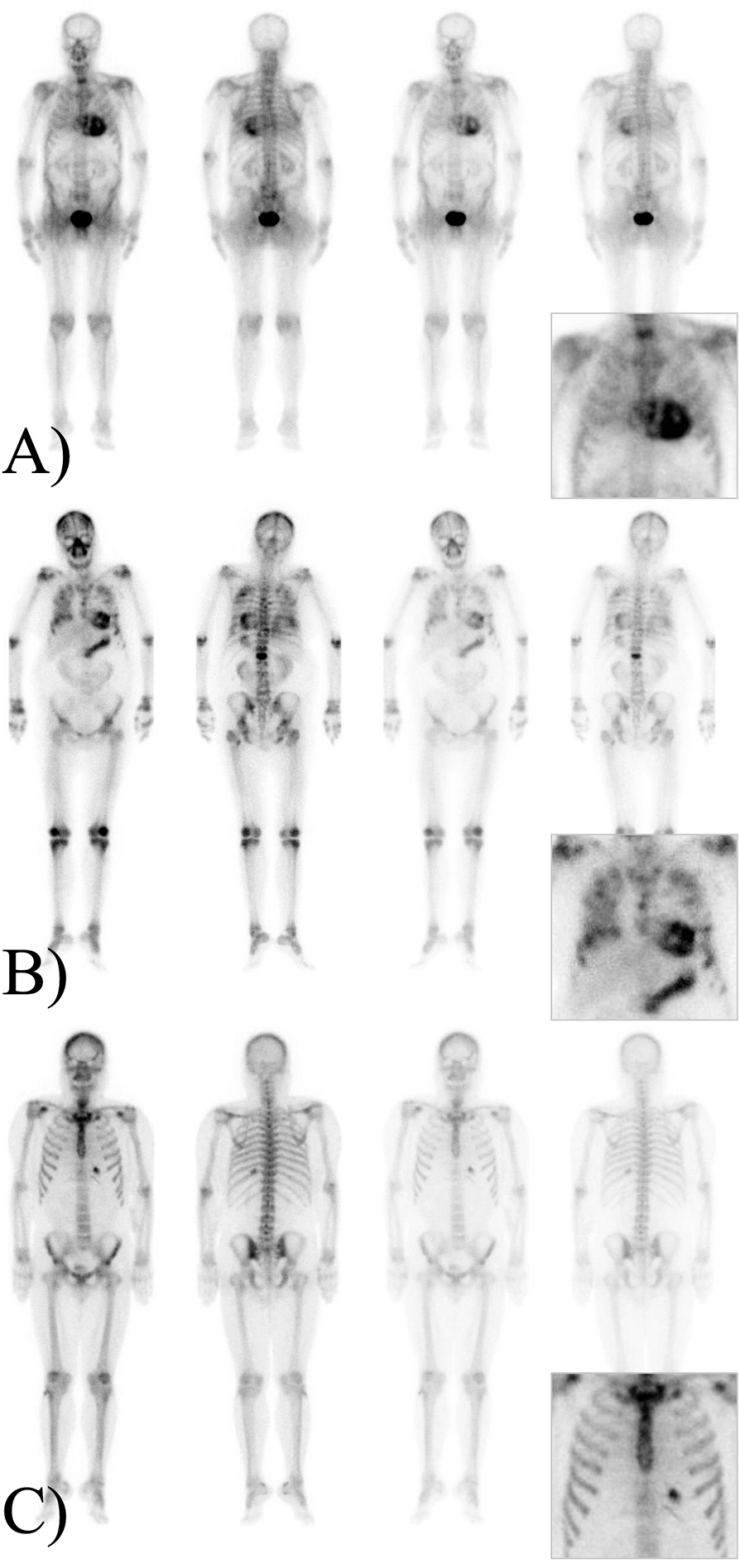
Representative cases of cardiac uptake in bone scintigraphy. **A** A 78-year-old male with grade 3 diffuse myocardial uptake, indicating transthyretin cardiac amyloidosis (ATTR-CA). **B** A 56-year-old female dialysis patient with metastatic breast cancer demonstrated grade 3 cardiac uptake due to metastatic calcification as well as multifocal increased osseous uptake as a result of bone metastasis. **C** A 51-year-old male patient on chronic dialysis with a history of myocardial infarction showed focally increased cardiac uptake. Inset boxes indicate a zoomed-in view of the heart and its surrounding bone structure

Cardiac uptake was classified as grade 2 if the tracer uptake in the myocardium was equal to that in the rib, and as grade 3 if the cardiac uptake was greater than that in the rib [9]. Patients with cardiac uptake were categorized into two groups. Patients with diffuse myocardial uptake of grade 2 or 3 were assigned to the possible ATTR-CA group (Fig. 2A) [9]. Patients having cardiac uptake for other obvious reasons, such as metastatic calcification associated with ESRD (Fig. 2B), or focal cardiac uptake that did not match cardiac amyloidosis (CA) (Fig. 2C), were included in the non-CA group. As a quantitative analysis, a circular region of interest (ROI) was drawn over the heart on the anterior image, copied, and mirrored on the contralateral chest. The heart to contralateral ratio was calculated as the ratio of the mean counts in the heart ROI to the contralateral chest ROI.

### Statistical analysis

Continuous variable data is given as mean ± standard deviation, whereas categorical variable data is expressed as frequencies. The Fisher exact test was used for between-group comparison of categorical variables. The numeric data were statistically compared using the Student t-test. The statistical analysis was conducted using IBM SPSS ver. 27 (IBM Corp). *P*-values less than 0.05 were deemed statistically significant.

## Results

### Characteristics of the entire population who underwent bone scintigraphy

The mean age of the 32,245 patients was 55 ± 17 years (range, 0–101 years) and 17,283 (53.6%) were male. Bone scintigraphy was performed on 16,961 patients (52.6%) for oncologic evaluation and 11,866 (36.8%) for orthopedic purposes. In the remaining 3,418 patients (10.6%), bone scintigraphy was performed for other indications such as evaluating metabolic bone disease in the presence of hypercalcemia. The percentage of bone tracers employed were 62.5% for ^99m^Tc-DPD and 37.5% for ^99m^Tc-HMDP.

### Study population and baseline characteristics

Of the 61,432 bone scintigraphic images acquired in 32,245 patients, 28 images from 23 patients (0.07%) showed grade 2 or 3 cardiac uptake, indicating the likelihood of ATTR-CA. The mean age of the 23 patients with cardiac uptake was 71 ± 18 years, and 13 (56.5%) were male. Sixteen patients (69.6%) had a history of hypertension and seven (30.4%) had a history of diabetes mellitus.

In 12 patients (52.2%), the indication of bone scintigraphy was cancer staging, and in 10 patients (43.5%), the indication was orthopedic problems such as fractures. Nineteen patients (91.3%) had bone scans only, and two patients (8.7%) had SPECT as an initial test; the SPECT scans of these patients did not include the chest because the purpose of the scan in both patients was a femur fracture. Two patients (8.7%) underwent SPECT as an additional test after showing cardiac uptake on the bone scans. Bone tracers, ^99m^Tc-DPD and ^99m^Tc-HMDP, were employed in 11 (47.8%) and 12 patients (52.2%), respectively. The proportion of patients with Perugini grade 2 and 3 uptake was 47.8% and 52.2%, respectively.

Atrial fibrillation on ECG was found in one patient (4.3%). LV hypertrophy by voltage criteria, low QRS voltage, and pseudoinfarct pattern were observed in seven (30.4%), two (8.7%), and four patients (17.4%), respectively. The mean level of N-terminal pro-brain natriuretic peptide (NT-proBNP) was 3,881 ± 5,952 pg/mL. The mean level of cardiac troponin T was 0.07 ± 0.04 ng/mL.

The mean LVEF of the 16 patients who underwent echocardiography was 51.5% ± 15.2%. The mean GLS was −12.9% ± 4.7%. The mean thickness of the LV posterior wall and interventricular septum was 10.2 ± 2.1 and 10.4 ± 2.0 mm, respectively. The mean anteroposterior diameter of the left atrium was 40.2 ± 6.9 mm and peak early transmitral ventricular filling velocity to early diastolic tissue Doppler velocity (E/e’) ratio was 15.7 ± 6.5.

### The possible ATTR-CA group vs. the non-CA group

Nine of 23 patients (39.1%) were classified as the non-CA group because they had a clear cause of cardiac uptake other than CA, such as metastatic calcification related to ESRD or metastatic cancer (Fig. 2B) or showed focal cardiac uptake inconsistent with the ATTR-CA (Fig. 2C). Six of the seven patients (85.7%) had metastatic calcification at sites other than the heart. The remaining 14 patients (60.9%) were assigned to the possible ATTR-CA group, of whom eight underwent echocardiography and five showed no evidence of monoclonal gammopathy. Five patients were referred to cardiologists and were eventually diagnosed with ATTR-CA. Two patients were treated with tafamidis (Fig. 1).

The mean age of the possible ATTR-CA group was significantly higher than the non-CA group (85 ± 3 years vs. 50 ± 10 years, *P* < 0.001). There was no significant difference in the distribution of sex between the two groups, nor the frequency of hypertension or a history of cancer or HF (Table 1). However, diabetes mellitus was more common in the non-CA group than in the possible ATTR-CA group (14.3% vs. 55.6%, *P* = 0.036). Furthermore, a history of ESRD was significantly more prevalent in the non-CA group than in the possible ATTR-CA group (0% vs. 66.7%, *P* < 0.001).

**Table 1:** Comparison of the characteristics between the possible ATTR-CA group and non-CA group

Variable	Overall patients with cardiac uptake (*n* = 23)	Possible ATTR-CA group (*n* = 14)	Non-CA group (*n* = 9)	*P*-value^a^
Clinical characteristic				
Age (yr)	71 ± 18	85 ± 3	50 ± 10	< 0.001^*^
Male sex	13 (56.5)	6 (42.9)	7 (77.8)	0.099
Past medical history				
Hypertension	16 (69.6)	9 (64.3)	7 (77.8)	0.493
Diabetes mellitus	7 (30.4)	2 (14.3)	5 (55.6)	0.036^*^
End-stage renal disease	6 (26.1)	0 (0)	6 (66.7)	< 0.001^*^
Heart failure	6 (26.1)	4 (28.6)	2 (22.2)	0.735
Cancer	16 (69.6)	11 (78.6)	5 (55.6)	0.242
Bone scintigraphic study				
Indication for study				0.052
Oncologic	12 (52.2)	10 (71.4)	2 (22.2)	
Orthopedic	10 (43.5)	4 (28.6)	6 (66.7)	
Other	1 (4.3)	0 (0)	1 (11.1)	
Cardiac uptake				0.049^*^
Grade 2	11 (47.8)	9 (64.3)	2 (22.2)	
Grade 3	12 (52.2)	5 (35.7)	7 (77.8)	
Heart to contralateral lung ratio	1.92 ± 0.77	2.00 ± 0.76	1.78 ± 0.81	0.514
Study technique as an initial test				0.235
Bone scan	21 (91.3)	12 (85.7)	9 (100)	
Bone SPECT^b^	2 (8.7)	2 (14.3)	0 (0)	
Bone tracer				0.265
^99m^Tc-DPD	11 (47.8)	8 (57.1)	3 (33.3)	
^99m^Tc-HMDP	12 (52.2)	6 (42.9)	6 (66.7)	
Electrocardiographic finding				
Atrial fibrillation	1 (4.3)	1 (7.1)	0 (0)	0.412
LVH by voltage criteria	7 (30.4)	5 (35.7)	2 (22.2)	0.493
Low QRS voltage	2 (8.7)	2 (14.3)	0 (0)	0.235
Pseudoinfarct pattern	4 (17.4)	3 (21.4)	1 (11.1)	0.524
Laboratory finding				
NT-proBNP (pg/mL)	3,881 ± 5,952	3,460 ± 6,052	8,509 ± 0	0.443
Troponin T (ng/mL)	0.07 ± 0.04	0.06 ± 0.04	0.10 ± 0.05	0.154
eGFR (mL/min/1.73 m^2^)	51.2 ± 36.1	75.5 ± 11.7	29.9 ± 37.2	0.010^*^
Echocardiographic finding				
LVEF (%)	51.5 ± 15.2	50.1 ± 17.2	53.3 ± 13.3	0.694
GLS (%)	−12.9 ± 4.7	−10.48 ± 3.63	−17.85 ± 0.07	0.054
LVPW thickness (mm)	10.2 ± 2.1	10.1 ± 2.3	10.3 ± 2.1	0.878
IVS thickness (mm)	10.4 ± 2.0	10.7 ± 2.1	10.1 ± 2.0	0.620
Left atrium diameter (mm)	40.2 ± 6.9	40.8 ± 6.8	39.4 ± 7.5	0.712
E/e’ ratio	15.7 ± 6.5	17.5 ± 7.6	13.5 ± 4.3	0.235

*ATTR-CA* Transthyretin cardiac amyloidosis, *CA* Cardiac amyloidosis, *SPECT* Single-photon emission computed tomography, ^*99m*^*Tc-DPD* Technetium-99m–labeled 3,3-diphosphono-1,2-propanodicarboxylic acid, ^*99m*^*Tc-HMDP* Technetium-99m–labeled hydroxymethylene diphosphonate, *LVH* Left ventricular hypertrophy, *NT-proBNP* N-terminal pro-brain natriuretic peptide, *eGFR* Estimated glomerular filtration rate, *LVEF* Left ventricular ejection fraction, *GLS* Global longitudinal strain, *LVPW* Left ventricular posterior wall, *IVS* Interventricular septum, *E/e’* Peak early transmitral ventricular filling velocity to early diastolic tissue Doppler velocity

^*^P < 0.05

^a^Comparison between the possible ATTR-CA group and non-CA group

^b^Including the heart

Indications of bone scintigraphy were dominated by oncologic purposes at 71.4% in the possible ATTR-CA group, while orthopedic indications were considerably more common at 66.7% in the non-CA group, with borderline statistical significance (*P* = 0.052). The initial test procedure between bone scan and SPECT, the bone tracer employed, the grades of cardiac uptake, and the heart to contralateral ratio were all comparable between the two groups (Table 1).

The distribution of ECG abnormalities such as atrial fibrillation, LV hypertrophy, low QRS voltage, and pseudoinfarct pattern was similar in both groups. Laboratory results showed comparable levels of NT-proBNP and cardiac troponin T (Table 1). However, the non-CA group had a considerably lower estimated glomerular filtration rate than the possible ATTR-CA group (75.5 ± 11.7 mL/min/1.73 m^2^ vs. 29.9 ± 37.2 mL/min/1.73 m^2^, *P* = 0.010).

Echocardiography was conducted on nine patients (64.3%) in the possible ATTR-CA group and seven patients (77.8%) in the non-CA group. The possible ATTR-CA group had worse LV systolic function (LVEF and GLS) and LV diastolic function (left atrium diameter and E/E’ ratio) than the non-CA group; however, none of these differences were statistically significant. The thickness of the LV posterior wall and interventricular septum were similar in both groups (Table 1).

### The rate of incidental ATTR-CA in the high-risk group

In this study, we arbitrarily defined the high-risk group for ATTR-CA as patients aged 75 years or older because the youngest patient in the possible CA group was 78 years old. Of the total 32,445 patients, 6,175 (19.2%) were aged 75 years or older. Their mean age was 80 ± 4 years (median, 79 years), and 59.5% were male. In this high-risk group, incidental cardiac uptake consistent with ATTR-CA was found in 14 patients (0.23%).

## Discussion

The key findings of this study are as follows: (1) Of the 32,245 patients who underwent 61,432 bone scintigraphic studies without HF symptoms at a tertiary teaching hospital for orthopedic indications or cancer staging, 23 (0.07%) showed cardiac uptake; (2) 14 of the 23 patients (60.9%) had cardiac uptake consistent with ATTR-CA (the possible ATTR-CA group), while the remaining nine patients (39.1%) were determined to have cardiac uptake not compatible with ATTR-CA (the non-CA group); (3) five patients (35.7%) in the possible ATTR-CA group were referred to cardiologists and confirmed to be wtATTR-CA; and (4) patients in the possible ATTR-CA group were significantly older, had a less frequent history of ESRD and diabetes mellitus, and had a higher estimated glomerular filtration rate than those in the non-CA group.

Several studies have been conducted to determine the prevalence of unintentionally detected ATTR-CA. Mohamed-Salem et al. [10] examined 1,509 scans from 1,114 patients aged 75 years and older who attended bone scintigraphy tests at a university hospital for orthopedic or oncologic reasons without suspicious findings of ATTR-CA throughout 7 years. Thirty-one patients (2.78%) had grade 2 or 3 cardiac uptake, and these patients were older and more likely to be male than those who did not have uptake. The prevalence of myocardial uptake in men aged 85 years and higher was as high as 13.9%. Patients who had cardiac uptake were hospitalized for HF at a considerably higher rate than those who did not (29% vs. 14%, *P* = 0.034). Bianco et al. [11] studied all patients (*n* = 4,228) who had bone scintigraphy at a university hospital for 5 years and discovered that ATTR-CA was present in 23 patients (0.54%). The most common reason for bone scintigraphy was oncologic indications (47.9%). Among the 23 patients, 11 (48.0%) had a history of HF, eight (34.8%) had carpal tunnel syndrome, five (21.7%) had neuropathy, and five (21.7%) were completely asymptomatic. Longhi et al. [7] analyzed all patients who underwent bone scintigraphy for oncologic or rheumatologic reasons over 5 years, excluding patients with suspected CA, and reported a frequency of myocardial uptake of 0.36% (45 of 12,400 patients; median age, 81 years [range, 65–82 years]; male sex, 62%), with prevalence increasing with age. One of the 45 patients had cardiac uptake due to bone metastasis; four patients (8.9%) with cardiac uptake had HF symptoms, and three (6.7%) had a previous history of carpal tunnel syndrome. Kim et al. [12] examined all patients who underwent bone scintigraphy with ^99m^Tc-DPD for noncardiac indications and were older than 30 years. Of the 9,581 patients enrolled in the study, the prevalence of positive cardiac uptake was 0.06%. All patients were older than 70 years, and the frequency of cardiac uptake in patients older than 70 years was only 0.4%.

In this current study, nine patients (39.1%) with cardiac uptake on bone scintigraphy exhibited either focal cardiac uptake or bone and soft tissue uptake in addition to the myocardium, rather than diffuse uptake in the myocardium, which is the diagnostic finding for ATTR-CA. It is well known that delayed or impaired renal excretion of radiotracers in ESRD patients results in increased bone and soft tissue uptake [13, 14]. In our study, six patients (66.7%) with non-CA cardiac uptake were due to metastatic calcifications associated with ESRD. Previous studies using a design similar to ours have not revealed an increase in myocardial uptake associated with metastatic calcifications in patients with ESRD in bone scintigraphy [7, 10, 11]. Bianco et al. [11] included all patients who presented for bone scintigraphy in their analysis. Kim et al. [12] did not describe the purposes of bone scintigraphy. Still, the remaining studies divided the indications of bone scintigraphy into two categories: rheumatologic and oncologic [7, 10]. In contrast, in our analysis, 10.6% of the entire study group and 4.3% of patients with cardiac uptake received bone scintigraphy to examine metabolic bone disease associated with hypercalcemia. Furthermore, none of the studies listed ESRD as one of the comorbidities. Our investigation found that individuals with ESRD-associated cardiac uptake had a mean age of 46 ± 10 years and 33% were female, which differed from the clinical features of ATTR-CA.

In comparison to previous studies, this research appears to have a relatively low prevalence of ATTR-CA (Table 2) [7, 10–12]. The study of Mohamed-Salem et al. [10] included only patients older than 75 years, given the predisposing population for ATTR-CA, and showed a prevalence of 2.78%, which was significantly higher than the other studies. In contrast, the patients in our study, including all those who underwent bone scintigraphy, were substantially younger, with a mean age of 55 ± 17 years, which may have contributed to the disparity in prevalence of ATTR-CA. In this study, cardiac uptake compatible with ATTR-CA was observed only in patients aged 78 years or older, and the prevalence of incidental ATTR-CA increased to 0.23% when we analyzed only the 6,175 patients aged 75 years or older, a high-risk group for wtATTR-CA. In this current study, orthopedic purposes and metabolic bone disease evaluations made up 47.4% of the indications of bone scintigraphy, while oncologic indications accounted for the majority at 95% to 95.7% of the patients in other studies [7, 10]. These differences may also have altered the features of patient populations included in the studies as well as the prevalence of ATTR-CA.

**Table 2:** Comparison of the studies that investigated the prevalence of incidental ATTR-CA in patients who underwent BS for indications other than ATTR-CA diagnosis

Characteristic	This study	Mohamed-Salem et al. [10] (2018)	Bianco et al. [11] (2021)	Longhi et al. [7] (2014)	Kim et al. [12] (2019)
No. of patients	32,245 (61,432 scans)	1,114 (1,509 scans)	4,228	12,400	9,580
Enrollment period	2011–2022	2010–2016	Jan 2015–May 2020	2008–May 2013	Jun 2014–Mar 2017
Country	Korea	Spain	Italy	Italy	Korea
Age (yr)	55 ± 17 (0–101)^a^	≥ 75	NA	74 (65–82)^a^	> 30
Male sex	53.6%	NA	NA	37%	NA
Enrollment criteria and indications of BS	All patients underwent BS not for evaluation of ATTR-CA (oncologic, 52.6%; orthopedic, 36.8%; other, 10.6%)	Patients underwent BS for oncologic (95.7%) and rheumatologic (4.3%) reasons without clinical suspicion of ATTR-CA	All-comers population who underwent a BS for any reasons	Patients underwent BS for oncologic (95%) or rheumatologic (5%) reasons without clinical suspicion of ATTR-CA	Patients underwent BS for noncardiac reasons
Bone tracer	^99m^Tc-DPD, 62.5% ^99m^Tc-HMDP, 37.5%	^99m^Tc-DPD, 13% ^99m^Tc-HDP, 40% ^99m^Tc-HMDP, 47%	^99m^Tc-HMDP, 82.9%	^99m^Tc-DPD, 100%	^99m^Tc-DPD, 100%
Positive cardiac uptake					
Definition	Grade 2 or 3	Grade 2 or 3	Grade 2 or 3	NA	Grade 2 or 3
Prevalence	23/32,245 (0.07%) ATTR-CA, 14/32,245 (0.04%)	31/1,114 (2.78%)	23/4,228 (0.54%)	45/12,400 (0.36%)	6/9,580 (0.06%)
Age (yr)^a^	71 ± 18 (ATTR-CA, 85 ± 3)	80.5 ± 4.1	83 ± 5	81 (77–84)	80.7 ± 7.5
Male sex	56.5% (ATTR-CA, 42.9%)	65%	78%	62%	83%
Cardiac uptake pattern	39.1% (9/23), not consistent with ATTR-CA	NA	NA	2.2% (1/45), localized uptake due to bone metastasis	NA
Clinical evidence of ATTR-CA	History of HF, 26.1%	History of HF, 19.3% Overall hospitalization for HF, 29.3% CTS, 16.0% Autonomic neuropathy, 6.4%	History of HF, 48.0% CTS, 34.8% Neuropathy, 21.7%	Dyspnea (NYHA Fc II), 8.9% (4/45) Pacemaker, 2.2% (1/45) CTS, 6.7% (3/45)	LVH, 66.7% (4/6) Diastolic dysfunction on echocardiography, 100% (6/6)

*ATTR-CA* Transthyretin cardiac amyloidosis, *BS* Bone scintigraphy, *NA* Not available, ^*99m*^*Tc-DPD* Technetium-99m–labeled 3,3-diphosphono-1,2-propanodicarboxylic acid, ^*99m*^*Tc-HMDP* Technetium-99m–labeled hydroxymethylene diphosphonate, ^*99m*^*Tc-HDP* Technetium-99–labeled hydroxyethylene diphosphonate, *HF* Heart failure, *CTS* Carpal tunnel syndrome, *NYHA* New York Heart Association, *Fc* Functional class, *LVH* Left ventricular hypertrophy

^a^Values are presented as mean ± standard deviation age (range), mean (range) or mean ± standard deviation

Despite the limited number of studies to compare, it is worth noting that the prevalence of incidental ATTR-CA in our study and Kim et al. [12]’s, both of which were conducted in Korea, is similarly low compared to other studies. They analyzed patients over the age of 30 years, and the prevalence of ATTR-CA was 0.06%. If we use the same inclusion criteria in our study, the prevalence of cardiac uptake was 0.08%, and the prevalence of ATTR-CA was 0.05%, which is still low in contrast to other studies. A main limitation of the studies examining the prevalence of incidental ATTR-CA is that almost all of them are retrospective. Therefore, the demographics of the patients in each study, such as age and sex, as well as the purpose of the bone scintigraphy, may have contributed to the differences in incidence. We cautiously speculate that the distinctly lower incidence in the two studies from Korea compared with studies from other (primarily European) countries could be attributable to the differences in demographic characteristics such as race, age, and sex of the populations included in each study or unidentified disparities between the healthcare systems in each country.

Because many patients with oncologic or orthopedic disorders are elderly and physically inactive, the symptoms of undiagnosed HF can be modest or concealed, leading to a delayed diagnosis. ATTR-CA is typically diagnosed late because symptoms are ambiguous or preceded by symptoms other than HF. However, a simple, noninvasive test called bone scintigraphy can now be used to confirm the diagnosis of ATTR-CA. Once diagnosed, there is an opportunity to improve the clinical trajectory of the disease with novel therapeutic agents such as tafamidis. wtATTR-CA is highly prevalent in elderly men, and if bone scintigraphy is conducted for purposes other than HF, such as orthopedic or cancer evaluation, careful attention to this may aid in the early detection of ATTR-CA. Clinical information is critical in reaching a diagnosis, as not all cases of cardiac uptake on bone scintigraphy are ATTR-CA, and other reasons must be addressed, particularly metastatic calcification in patients with ESRD. Nuclear imaging specialists reading the bone scintigraphy should not overlook the presence of myocardial uptake, even if the scan is not for HF, particularly in older, high-risk patients. The physician should refer the patient with cardiac uptake to a cardiologist for further assessment and treatment for ATTR-CA. In our study, only about one-third of patients with cardiac uptake received further evaluation for ATTR-CA.

There are several limitations in our study. First, this is a retrospective study that was not designed to establish the prevalence of ATTR-CA in all patients having bone scintigraphy, hence the prevalence of ATTR-CA is likely to be underestimated. Second, only a subset of patients with cardiac uptake completed the diagnostic testing for ATTR-CA such as echocardiography and monoclonal gammopathy screening. Third, cardiac uptake may be overlooked in patients undergoing bone scintigraphy for reasons other than the diagnosis of ATTR-CA if cardiac uptake is not thoroughly investigated. The prevalence would have been much higher if all bone scintigraphy images from all patients had been reviewed for probable missed cardiac uptake, but this was not feasible due to the large number of patients and images. Fourth, only 15.8% of patients with cardiac uptake received bone SPECT. Patients with cardiac uptake on bone scans were included in the study if it was determined to be cardiac uptake by careful review of the images by two imaging cardiologists, although it is possible that there was spurious cardiac uptake in patients who did not undergo SPECT. Lastly, although recent studies and statements have suggested that histologic diagnosis should be considered in patients with Perugini grade 1 uptake, our study did not further investigate grade 1 uptake due to the large number of patients [15, 16].

## Conclusions

Bone scintigraphic studies are performed for a wide range of indications, and patients undergoing bone scintigraphy for orthopedic and oncologic purposes, which account for the vast majority of indications, may have poor performance status, masking symptoms of HF. Although incident cardiac uptake on bone scintigraphy is quite uncommon, the presence of cardiac uptake, particularly in elderly patients without evidence of metastatic calcification associated with ESRD, should prompt a diagnostic evaluation for ATTR-CA, which is an undiagnosed and treatable cause of HF.

## Data Availability

Not applicable.

## Abbreviations

^99^^m^Tc-DPD: Technetium-99m–labeled 3,3-diphosphono-1,2-propanodicarboxylic acid
^99^^m^Tc-HMDP: Technetium-99m–labeled hydroxymethylene diphosphonate
ATTR-CA: Transthyretin cardiac amyloidosis
CA: Cardiac amyloidosis
ECG: Electrocardiogram
E/e’: Peak early transmitral ventricular filling velocity to early diastolic tissue Doppler velocity
ESRD: End-stage renal disease
GLS: Global longitudinal strain
HF: Heart failure
LA: Left atrium
LV: Left ventricular
LVEF: Left ventricular ejection fraction
NT-proBNP: N-terminal pro-brain natriuretic peptide
ROI: Region of interest
SPECT: Single-photon emission computed tomography
wtATTR-CA: Wild-type transthyretin cardiac amyloidosis
